# Supplementary material for: Active site specificity profiling datasets of matrix metalloproteinases (MMPs) 1, 2, 3, 7, 8, 9, 12, 13 and 14
Source: Data Brief. 2016 Feb 22;7:299–310. doi: 10.1016/j.dib.2016.02.036 (PMC4777984; doi:10.1016/j.dib.2016.02.036)
Supplement: Supplementary file 10 — Supplementary material [file mmc10.zip › WebPICS_hMMP14_T_1%/P3prime.html]

 

PICS results


|  |  |
| --- | --- |
| **P3prime\_D**  17 in 307 sites   5.5 %    effects > 10 perc. pnts.   (vice-versa in brackets)  P2\_Q: 22.6 (18.3) |  |
| **P3prime\_L**  33 in 307 sites   10.7 %    effects > 10 perc. pnts.   (vice-versa in brackets)  P1\_N: 21.2 (19.0) |  |
| **P3prime\_N**  16 in 307 sites   5.2 %    effects > 10 perc. pnts.   (vice-versa in brackets)  P1\_C: 10.2 (23.4) |  |
| **P3prime\_Q**  13 in 307 sites   4.2 %    effects > 10 perc. pnts.   (vice-versa in brackets)  P2prime\_C: 21.1 (45.8) |  |
| **P3prime\_S**  26 in 307 sites   8.5 %    effects > 10 perc. pnts.   (vice-versa in brackets)  P1\_P: 14.0 (12.9) |  |
| **P3prime\_V**  26 in 307 sites   8.5 %    effects > 10 perc. pnts.   (vice-versa in brackets)  P3\_S: 12.4 (15.3)   P2prime\_M: 10.5 (18.2) |  |
